# Supplementary material for: A Novel Fiber-Optic Ice Sensor to Identify Ice Types Based on Total Reflection
Source: Sensors (Basel). 2023 Apr 14;23(8):3996. doi: 10.3390/s23083996 (PMC10145984; doi:10.3390/s23083996)
Supplement: Supplementary file 1 [file sensors-23-03996-s001.zip › sensors-2295016-supplementary.pdf]

Simulation and experimental results

The relationship between the light intensity received by the sensor and the ice thickness during the simulation.

Table S1. Simulation of glazed ice.

glazed ice thickness(mm)	optical Intensity(RF1)(mW)	optical Intensity(RF2)(mW)	optical Intensity(RF3)(mW)
0.0	0.00000000	0.00000000	0.00000000
0.5	0.00003157	0.00000000	0.00000000
1.0	0.00008785	0.00000000	0.00000000
1.5	0.00013059	0.00000266	0.00000000
2.0	0.00012005	0.00000495	0.00000000
2.5	0.00009753	0.00002320	0.00000000
3.0	0.00007784	0.00003874	0.00000000
3.5	0.00006127	0.00003850	0.00000000
4.0	0.00004726	0.00003662	0.00000000
4.5	0.00003450	0.00003418	0.00000000
5.0	0.00002791	0.00003157	0.00000000

Table S2. Simulation of rime ice1.

rime ice1 thickness(mm)	optical Intensity(RF1)(mW)	optical Intensity(RF2)(mW)	optical Intensity(RF3)(mW)
0.0	0.00000000	0.00000000	0.00000000
0.5	0.00002343	0.00000249	0.00000000
1.0	0.00006510	0.00001692	0.00000071
1.5	0.00007285	0.00003010	0.00000141
2.0	0.00007177	0.00004081	0.00000215
2.5	0.00007958	0.00004663	0.00000319
3.0	0.00008018	0.00005135	0.00000388
3.5	0.00008343	0.00005554	0.00000458
4.0	0.00008683	0.00005631	0.00000458
4.5	0.00008817	0.00005812	0.00000458
5.0	0.00009010	0.00005954	0.00000529

Table S3. Simulation of rime ice2.

rime ice2 thickness(mm)	optical Intensity(RF1)(mW)	optical Intensity(RF2)(mW)	optical Intensity(RF3)(mW)
0.0	0.00000000	0.00000000	0.00000000
0.5	0.00020422	0.00000905	0.00000790
1.0	0.00038313	0.00003579	0.00001957
1.5	0.00046222	0.00006134	0.00002728
2.0	0.00049775	0.00007795	0.00003054
2.5	0.00051147	0.00009245	0.00003231
3.0	0.00051892	0.00009814	0.00003264

3.5	0.00052194	0.00010126	0.00003369
4.0	0.00052417	0.00010529	0.00003406
4.5	0.00052707	0.00010603	0.00003512
5.0	0.00052768	0.00010772	0.00003548

The relationship between the voltage output by the sensor and the ice thickness during the experiment.

Table S4. The experiment of glazed ice.

glazed ice thickness(mm)	Out voltage(RF1)(V)	Out voltage (RF2)(V)	Out voltage (RF3)(V)
0.000	0.0000	0.0000	0.0000
0.512	0.0151	0.0000	0.0012
0.989	0.0294	0.0000	0.0012
1.524	0.0627	0.0059	0.0012
1.991	0.0836	0.0098	0.0013
2.472	0.0725	0.0214	0.0013
3.102	0.0675	0.0279	0.0011
3.564	0.0517	0.0267	0.0013
4.091	0.0408	0.0258	0.0012
4.493	0.0340	0.0247	0.0011
5.017	0.0257	0.0234	0.0013

Table S5. The experiment of rime ice1.

rime ice1 thickness(mm)	Out voltage(RF1)(V)	Out voltage (RF2)(V)	Out voltage (RF3)(V)
0.000	0.0000	0.0000	0.0000
0.491	0.0591	0.0051	0.0020
1.098	0.0690	0.0084	0.0020
1.476	0.0843	0.0181	0.0021
1.987	0.0859	0.0280	0.0021
2.563	0.0867	0.0291	0.0021
2.987	0.0979	0.0380	0.0023
3.486	0.1017	0.0485	0.0023
4.067	0.1112	0.0538	0.0023
4.476	0.1190	0.0632	0.0023
5.058	0.1261	0.0682	0.0023

Table S6. The experiment of rime ice2.

rime ice2 thickness(mm)	Out voltage(RF1)(V)	Out voltage (RF2)(V)	Out voltage (RF3)(V)
0.000	0.0000	0.0000	0.0000
0.537	0.4013	0.0134	0.0060
1.093	0.524	0.0366	0.0071
1.554	0.5333	0.0658	0.0081
2.103	0.544	0.1145	0.0081

2.495	0.5582	0.1307	0.008
2.975	0.5720	0.1575	0.0087
3.548	0.5819	0.1698	0.0088
4.074	0.5950	0.1814	0.0091
4.564	0.6035	0.1990	0.0091
5.116	0.6109	0.2019	0.0090
